# Expanding the data Ark: an attempt to make the data from highly cited social science papers publicly available

**DOI:** 10.1098/rsos.240016

**Published:** 2024-05-15

**Authors:** Coby Dulitzki, Steven Michael Crane, Tom E. Hardwicke, John P. A. Ioannidis

**Affiliations:** ^1^ Department of Biology, Stanford University, Stanford, CA, USA; ^2^ Meta-Research Innovation Center at Stanford (METRICS), Stanford University, Stanford, CA, USA; ^3^ Departments of Medicine, of Epidemiology and Population Health, of Biomedical Data Science, and of Statistics, Stanford University, Stanford, CA, USA; ^4^ Stanford Prevention Research Center, Stanford School of Medicine, Stanford, CA, USA; ^5^ School of Psychological Sciences, University of Melbourne, Melbourne, Australia

**Keywords:** metaresearch, reproducibility, open science, data transparency, open data, social science

## Abstract

Access to scientific data can enable independent reuse and verification; however, most data are not available and become increasingly irrecoverable over time. This study aimed to retrieve and preserve important datasets from 160 of the most highly-cited social science articles published between 2008–2013 and 2015–2018. We asked authors if they would share data in a public repository—the Data Ark—or provide reasons if data could not be shared. Of the 160 articles, data for 117 (73%, 95% CI [67%–80%]) were not available and data for 7 (4%, 95% CI [0%–12%]) were available with restrictions. Data for 36 (22%, 95% CI [16%–30%]) articles were available in unrestricted form: 29 of these datasets were already available and 7 datasets were made available in the Data Ark. Most authors did not respond to our data requests and a minority shared reasons for not sharing, such as legal or ethical constraints. These findings highlight an unresolved need to preserve important scientific datasets and increase their accessibility to the scientific community.

## 1. Introduction

Sharing data can enable independent reuse and verification, thereby enhancing the efficiency, credibility, and reproducibility of scientific research [1,2]. However, most primary datasets are not published alongside the articles that draw on them, nor provided when requested from the original authors [3,4]. Despite calls for increased openness [5,6], adoption of transparent research practices in the social sciences remains low: one study estimated the field-wide prevalence of articles linked to immediately available primary data to be just 2% for articles published in 2014–2017 [7].

The present study is a continuation of a previous initiative in which we attempted to retrieve data from influential psychology and psychiatry articles and make them available to the scientific community in an unrestricted manner in a public online repository called the ‘Data Ark' [8]. Data from influential studies are especially important to preserve before they meet the fate of most scientific datasets: increasing inaccessibility and eventual irrecoverable loss [4,9]. Highly-cited studies largely shape the literature at large, so the loss of their data may be more consequential than the loss of data for studies that have a smaller influence on the literature. We do recognize nevertheless that data sharing is not always straightforward. Therefore, an additional aim of the present study is to learn about barriers to data sharing, such as legal or ethical constraints [10].

In the original Data Ark project, we sent data sharing requests to the authors of the 111 most highly cited studies in psychology and psychiatry published between 2006–2011 and 2014–2016. We asked if authors would be willing to make the data available, potentially in the Data Ark repository, noting that we were also interested in learning about why data could not be shared, should that be the case. Ultimately, data from 15 articles were provided in an unrestricted form. Of these 15, five already had a system for sharing the data and data from the other 10 articles were sent to us by the authors and made publicly available in the Data Ark (https://osf.io/meetings/DataArk). As of 29th June 2023, the 10 datasets in the Data Ark have been downloaded 343 times (range: 5–111).

In the present study, we sought to expand the Data Ark by attempting to retrieve, preserve, and liberate data from 160 of the most highly-cited empirical articles published in the social sciences. Social sciences were broadly defined according to the Clarivate Analytics Essential Science Indicators classification schema (https://shorturl.at/egkvF). To examine if there was any change in data availability over time, we selected articles published in two separate time frames: 2008–2013 and 2016–2018. We also stratified the articles between medical (e.g. epidemiology or public health) and non-medical domains, to compare data sharing practices in these two subsets of the social sciences.

For each of the 160 articles, we checked whether the data were already available according to statements in the article itself. If data were not available, we contacted the authors and invited them to share the data in the Data Ark, state whether the data could be made available in some restricted form, or provide reasons why the data could not be made available. The goal of this effort was to improve our understanding of data sharing practices, including how they may differ between medical and non-medical domains and how they may have changed over time. We also aimed to preserve data from some of the most highly-cited articles published in the social sciences, and make those data available for re-use and verification by the wider scientific community.

## 2. Methods

The study protocol (rationale, methods, and analysis plan) was pre-registered on June 20, 2019 (https://osf.io/697wx). All departures from this protocol are explicitly acknowledged in Appendix B.

### 2.1. Design

The study had a cross-sectional design.

### 2.2. Sample

Citation information was exported for the most cited articles in the ‘Social Sciences, General' research domain as identified by Clarivate Analytics Essential Science Indicators for 2008–2013 (*n* = 4670) and 2016–2018 (*n* = 2975). The search was performed on February 27, 2019. These represent the top 1% most-cited articles in the social sciences category, accounting for year of publication. The ‘Social Sciences, General' category is very broad and it includes a large number of subfields. We aimed to have good representation of both medical subfields (those that have relevance to public health and/or social/population aspects of medicine) and non-medical subfields. Articles included in PubMed with a PMID number were classified as medical; other articles were classified as non-medical.

160 articles reporting on distinct primary datasets were identified by screening titles, abstracts, and, where necessary, full texts. Articles had to be written in the English language and have an accessible full-text version. We aimed to identify 40 medical (have a PMID listed) and 40 non-medical (no PMID listed) articles for each time period (2008–2013 and 2016–2018). Screening proceeded in descending order of citation count until 40 in each of the four categories above were identified. Due to researcher error in the original sampling assignment, we mistakenly included 38 articles in the nonmedical 2008–2013 category, and 42 in the nonmedical 2016–2018 category.

### 2.3. Procedure and measured variables

We checked the 160 articles for a data availability statement or supplemental files containing the primary dataset. Of these, 14 articles provided datasets within or alongside the original publication and 146 lacked publicly available datasets. The corresponding authors for these 146 articles were emailed and invited to share their data to be uploaded to the Data Ark on the Open Science Framework (see email template in Appendix). If we received a message stating that the email address was no longer functional, we searched for up-to-date contact information for the corresponding author and re-sent the initial request. Two reminder emails were sent at 2-week intervals to all non-responders. An initial test batch of 16 randomly selected authors was sent out on November 4th, 2019. The remaining requests were sent on November 11th, 2019. Ultimately, in all cases we managed to send an email to corresponding authors that did not bounce back, suggesting we had reached a functioning email account. We did not contact any other members of the research team or any sponsors.

### 2.4. Analysis

The data sharing status of each article was characterized according to the following categories (based on [8]):

— Article has a data sharing statement that says data are available
— No response to data sharing request
— Authors shared data for unrestricted sharing in the Data Ark
— Authors shared data for restricted sharing in the Data Ark
— Authors shared data only with us
— Authors stated that they will not share the data
— Authors stated that they are locating/preparing the data
— Authors stated that they are considering our data sharing request
— Authors stated that they require more information before they can share data
— Authors stated that they already have an unrestricted data sharing system already in place
— Authors stated that they already have a restricted data sharing system already in place

We additionally classified each article's data sharing status according to a higher-level schema as follows:

— Data available (unrestricted)
— Data available (restricted)
— Data not available

Counts and percentages to describe the data sharing status of the sampled articles are reported. We also report confidence intervals and Fisher's exact tests of differences between domains (medical versus non-medical) and between time periods (2008–2013 versus 2015–2018).

## 3. Results

### 3.1. Article characteristics

The sample of 160 articles comprised the most highly-cited English-language studies published in medical and non-medical subdomains of the social sciences between 2008–2013 and 2016–2018 (table 1).

**Table 1.** Article citations according to Web of Science as of February 27, 2019.

domain	time period	citations, median (interquartile range)
medical	2008–2013	514 (431–806)
medical	2016–2018	60 (52–84)
non-medical	2008–2013	388 (347–486)
non-medical	2016–2018	48 (44–67)

### 3.2. Data availability overall, between domains, and across time

**Figure 1.**
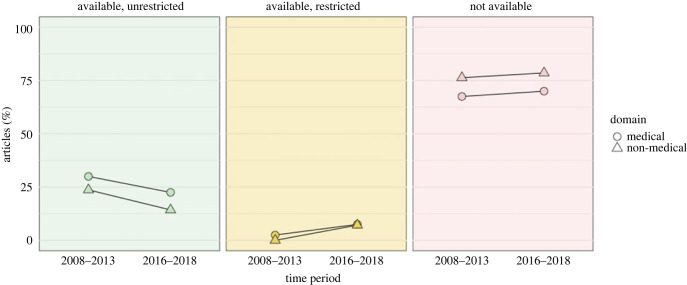
Data availability status for highly-cited articles in medical and non-medical domains of the social sciences across two time periods.

Overall, out of the 160 empirical articles, the data of 36 (22%, 95% CI [16%–30%]) were available in unrestricted form, the data of 7 (4%, 95% CI [0%–12%]) were available with restrictions, and the data of 117 (73%, 95% CI [67%–80%]) were not available. The differences between domains and between time periods were marginal (figure 1): compared to the datasets from the 2008–2013 articles, there were trends for proportionally fewer datasets available without restrictions, more available with restrictions, and more unavailable among the 2016–2018 articles. Datasets in the medical domain were available slightly more often than datasets in the non-medical domain. Fisher's exact tests indicated no statistically significant association between data sharing status and time period (two-tailed *p* = 0.098) or domain (two-tailed *p* = 0.447).

### 3.3. Data available without restrictions

Of the 36 datasets available in unrestricted form, 14 were from articles which contained a data availability statement providing a link to the available data; 15 were from articles that did not contain a data availability statement, but the authors informed us that there was an unrestricted data sharing system already in place; and 7 were from articles for which the authors agreed to make the data freely available in the Data Ark (see https://osf.io/ndmsr/). In all of these cases, we verified that the data were actually available.

### 3.4. Data available with restrictions

Of the seven datasets available with restrictions, one was shared only with our team and the authors stated that they did not want the data to be shared publicly in the Data Ark. For the other six datasets, the authors informed us that there was a restricted data sharing system already in place. Two datasets required a fee for access. The other four datasets each had multiple restrictions. Three required a data use agreement. Two required approval from an ethics board. Four required sharing a specific intention for reusing the data. Three required that requestors be from a recognized academic institution. Two would only allow remote access to the data. We did not attempt to verify whether complying with these restrictions would enable us to access the data.

### 3.5. Data not available

Of the 117 datasets classified as unavailable, 93 involved no response from the authors, despite sending multiple reminders. In 3 cases, the authors said they were locating or preparing the data, but did not supply any data or respond to any follow up as of the time of publication since our most recent request on January 5th, 2021. For one of these cases, we may be responsible for the non-receipt of data as we couldn't immediately identify a way to transfer large data files, and this introduced a substantial delay in communications with an author. In the remaining 21 cases, the authors explicitly stated that they would not share the data. All of these authors provided at least one reason for not sharing the data. These were (a) legal or ethical constraints (*n* = 11); (b) data do not belong to the authors (*n* = 10); (c) lack of resources needed to prepare the data for sharing (*n* = 3); (d) the data no longer exist or cannot be located (*n* = 3); (e) the authors wish to retain exclusive use of the data (*n* = 1).

## 4. Discussion

We sought to retrieve, preserve, and make widely available the data underlying highly-cited studies published in the social sciences by asking authors if they would be willing to make the data available in an unrestricted public online repository—the Data Ark. Our efforts were largely unsuccessful; the modal response to our data sharing requests was no response (*n* = 93), and by the end of our study, 117 out of the 160 (73%) targeted datasets remained unavailable. Seven authors agreed to make previously unavailable data publicly available in the Data Ark. We observed no significant differences between time periods (2008–2013 versus 2016–2018) and subdomains of the social sciences (medical versus non-medical).

Our findings are consistent with other studies reporting challenges retrieving scientific data via author requests [4,9], including our prior Data Ark project, which focused on highly-cited psychiatry and psychology studies [8]. The obvious conclusion from these efforts is that we cannot presently assume data will be made available on request. A strategy that has been endorsed by the International Committee of Medical Journal Editors and which is becoming more common over time is to require that articles contain data availability statements upon publication, which either directly link to data, or explain what might be the future plan for sharing or why they cannot be shared [11,12]. Unfortunately, such forward-facing policies cannot address the lack of data sharing in already published articles, regardless of whether they may or may not be able to improve data sharing in the future. Preliminary evidence suggests that even with data sharing statements, the actual sharing of data and code remains uncommon [13,14]. A greater paradigm shift is needed to enable wider data sharing [15,16].

We recognize that data sharing is not always straightforward; an additional motivation for this project was to surface reasons why data cannot be shared. Unfortunately, only 21 authors provided such reasons and with such sparse data it is difficult to draw strong inferences. Some commonly mentioned constraints were ethical or legal; for example, some authors were concerned that data, especially sensitive personal data, would be misused or misrepresented by a third-party. Sometimes such constraints may be deemed insurmountable and public data sharing would not be appropriate. However, under some conditions full or partial sharing of potentially sensitive data is possible, either through the use of deanonymization tools or through a careful weighing of the ethical pros and cons [10,17]. Participant surveys suggest that they are usually in favour of wider sharing [18]. Minimally, we suggest that ethical or legal constraints are stated explicitly in data availability statements. Another common constraint was that data do not belong to the authors. This was also the most common constraint in our previous effort to create a Data Ark for the data from the most highly-cited psychology and psychiatry papers. [8] When possible, authors should seek permission from the data provider to share the data with the scientific community. If this is not possible, the data availability statement should at least clarify how the data were obtained. It is unclear whether lack of sharing would be justified in these circumstances. A few authors said that data were lost or could no longer be accessed, for example, because one individual was responsible for the data but could not be contacted. This highlights the dangers of relying on informal data management systems; storing data in a robust public online repository removes the dependency on any single person or computer system and reduces the possibility of data loss.

Encouragingly, data for 29 articles were already available in unrestricted form. However, only 14 of these articles were found to explicitly state this, whereas the shared data for the other 15 articles only became apparent when we contacted authors with our request. This again reinforces the need for articles to include explicit data availability statements with clear data access instructions. Of course, if additional data can become available downstream, this is welcome even if it had not been specified in the original publication. Current indexing systems do not offer updated information on the status of sharing data for each publication; this may be a feature that could be enhanced: e.g. allowing authors in PubMed to provide links to newly released datasets.

The data for an additional seven articles is now safely preserved and publicly available in the Data Ark (https://osf.io/ndmsr/). It would be interesting to track how often these datasets are accessed. Our experience from similar datasets released for most-cited psychology and psychiatry articles showed that some of them are accessed often while others are only minimally accessed. Experience from clinical trials' data platforms suggest that many requests are made, about half of them are approved, and at least a sixth lead to some publication(s), mostly secondary analyses [19]. The quality of these secondary analyses is another important consideration as criticisms have emerged regarding data mining Large-Scale Volunteer Databases (LSVD) which has resulted in poor quality analyses and publications [20].

A further seven datasets were available in principle, but only if certain restrictions were adhered to, including paying an access fee or signing a data use agreement. When restrictions are necessary, it may be best if they are administered by a third-party data steward (such as the Yoda Project) who can independently adjudicate between the access needs of the scientific community and legitimate sharing constraints [21].

Our study had several limitations. Firstly, it is possible that various characteristics of our email request may have affected the study outcomes. For example, authors may have been more inclined to share data if we had outlined a specific research use for the data, other than the goal of preserving the data and making it available to others. Secondly, we did not attempt to adhere to the various requirements some authors placed on data access; it is therefore unknown if some of these data might be accessible in practice, if one were to request them for a specific project and followed a number of additional approval steps. Furthermore, we did not attempt to assess in detail whether all relevant study data were shared, whether data were properly documented, or whether the data could actually be used to reproduce the reported results. Prior studies have shown that simply having access to data does not guarantee that it is complete or understandable, or enables reproducibility [11,22,23]. The results of original publications may or may not be reproduced upon re-analysis [24–26]. Finally, the sampled category of ‘Social Sciences, General' encompasses a very large number of very different subfields beside the broad categorization into two domains (medical and non-medical). We cannot exclude the possibility that some subfield(s) may be more open to sharing than others. Our sample size was too small to explore such potential differences.

Data transparency is important to both validate findings as well as create opportunities for reuse in future studies. A range of efforts are underway to improve data access by prompting or incentivizing data sharing and making sharing easier through software tools and data repositories. Recent findings have indicated a modest increase in data sharing in the late 2010s, at least in biomedicine [3]; however, there remains considerable room for improvement. Unfortunately, prospective efforts to improve data sharing in future research are unlikely to improve retroactive access to data sets for studies that have already been published, and there is a danger that much important data will be lost. If authors can be encouraged to engage, initiatives like the Data Ark may help retrieve, preserve, and liberate valuable datasets of influential work to the benefit of the scientific community.

## Data Availability

All data exclusions and measures conducted during this study are reported in this manuscript. All data, materials, and analysis scripts are publicly available on the Open Science Framework (https://osf.io/3kuwq/ [27]).

## Code availability

To facilitate reproducibility the analysis script underlying the results reported in this manuscript is available in a Code Ocean container (https://doi.org/10.24433/CO.5136309.v2 [28]) which re-creates the software environment in which the original analyses were performed.

## References

[1] Doshi P, Goodman SN, Ioannidis JPA. 2013 Raw data from clinical trials: Within reach? Trends Pharmacol. Sci. **34**, 645-647. (10.1016/j.tips.2013.10.006) 24295825

[2] Klein O et al. 2018 A practical guide for transparency in psychological science. Collabra: Psychology **4**, Article 1. (10.1525/collabra.158)

[3] Serghiou S, Contopoulos-Ioannidis DG, Boyack KW, Riedel N, Wallach JD, Ioannidis JPA. 2021 Assessment of transparency indicators across the biomedical literature: How open is open? PLoS Biol. **19**, e3001107. (10.1371/journal.pbio.3001107) 33647013 PMC7951980

[4] Vines TH et al. 2014 The availability of research data declines rapidly with article age. Current Biology **24**, 94-97. (10.1016/j.cub.2013.11.014) 24361065

[5] Miguel E et al. 2014 Promoting transparency in social science research. Science **343**, 30-31. (10.1126/science.1245317) 24385620 PMC4103621

[6] Munafò, M. R. et al. (2017). A manifesto for reproducible science. Nature Human Behaviour **1** Article 1. 10.1038/s41562-016-0021 PMC7610724 33954258

[7] Hardwicke TE, Wallach JD, Kidwell MC, Bendixen T, Crüwell S, Ioannidis JPA. 2020 An empirical assessment of transparency and reproducibility-related research practices in the social sciences (2014–2017). R. Soc. Open Sci. **7**, 190806. (10.1098/rsos.190806) 32257301 PMC7062098

[8] Hardwicke TE, Ioannidis JPA. 2018 Populating the Data Ark: An attempt to retrieve, preserve, and liberate data from the most highly-cited psychology and psychiatry articles. PLoS ONE **13**, e0201856. (10.1371/journal.pone.0201856) 30071110 PMC6072126

[9] Minocher R, Atmaca S, Bavero C, McElreath R, Beheim B. 2021 Estimating the reproducibility of social learning research published between 1955 and 2018. R. Soc. Open Sci. **8**, 210450. (10.1098/rsos.210450) 34540248 PMC8441137

[10] Meyer MN. 2018 Practical Tips for Ethical Data Sharing. Advances in Methods and Practices in Psychological Science **1**, 131-144. (10.1177/2515245917747656) PMC6544443 31157320

[11] Hardwicke TE et al. 2018 Data availability, reusability, and analytic reproducibility: Evaluating the impact of a mandatory open data policy at the journal Cognition. R. Soc. Open Sci. **5**, 180448. (10.1098/rsos.180448) 30225032 PMC6124055

[12] Nuijten MB, Borghuis J, Veldkamp CLS, Dominguez-Alvarez L, Van Assen MALM, Wicherts JM. 2017 Journal data sharing policies and statistical reporting inconsistencies in psychology. Collabra: Psychology **3**, 31. (10.1525/collabra.102)

[13] Naudet F et al. 2021 Medical Journal Requirements for clinical trial data sharing: Ripe for improvement. PLoS Med. **18**, e1003844. (10.1371/journal.pmed.1003844) 34695113 PMC8575305

[14] Danchev V, Min Y, Borghi J, Baiocchi M, Ioannidis JPA. 2021 Evaluation of data sharing after implementation of the International Committee of Medical Journal Editors Data Sharing Statement Requirement. JAMA Network Open **4**, e2033972. (10.1001/jamanetworkopen.2020.33972) 33507256 PMC7844597

[15] Pellen C et al. 2023 Ten (not so) simple rules for clinical trial data-sharing. PLoS Comput. Biol. **19**, e1010879. (10.1371/journal.pcbi.1010879) 36893146 PMC9997951

[16] Mansmann U et al. 2023 Implementing clinical trial data sharing requires training a new generation of biomedical researchers. Nat. Med. **29**, 298-301. (10.1038/s41591-022-02080-y) 36732626

[17] Ross MW, Iguchi MY, Panicker S. 2018 Ethical aspects of data sharing and research participant protections. American Psychologist **73**, 138-145. (10.1037/amp0000240) 29481107

[18] Mello MM, Lieou V, Goodman SN. 2018 Clinical trial participants' views of the risks and benefits of data sharing. New England Journal of Medicine **378**, 2202-2211. (10.1056/nejmsa1713258) 29874542 PMC6057615

[19] Vazquez E, Gouraud H, Naudet F, Gross CP, Krumholz HM, Ross JS, Wallach JD. 2021 Characteristics of available studies and dissemination of research using major clinical data sharing platforms. Clinical Trials **18**, 657-666. (10.1177/17407745211038524) 34407656 PMC8595516

[20] Brayne C, Moffitt TE. 2022 The limitations of large-scale volunteer databases to address inequalities and global challenges in health and aging. Nature Aging **2**, 775-783. (10.1038/s43587-022-00277-x) 37118500 PMC10154032

[21] Lewandowsky S, Bishop D. 2016 Research integrity: Don't let transparency damage science. Nature. **529**, 459-461. (10.1038/529459a) 26819029

[22] Hardwicke TE et al. 2021 Analytic reproducibility in articles receiving open data badges at the journal Psychological Science: An observational study. R. Soc. Open Sci. **8**, 201494. (10.1098/rsos.201494) 33614084 PMC7890505

[23] Towse JN, Ellis DA, Towse AS. 2020 Opening Pandora's Box: Peeking inside psychology's data sharing practices, and seven recommendations for change. Behav. Res. Methods **53**, 1455-1468. (10.3758/s13428-020-01486-1) 33179123 PMC8367918

[24] Bergeat D, Lombard N, Gasmi A, Le Floch B, Naudet F. 2022 Data Sharing and reanalyses among randomized clinical trials published in surgical journals before and after adoption of a data availability and reproducibility policy. JAMA Network Open **5**, e2215209. (10.1001/jamanetworkopen.2022.15209) 35653153 PMC9163999

[25] Siebert M, Gaba J, Renault A, Laviolle B, Locher C, Moher D, Naudet F. 2022 Data-sharing and re-analysis for main studies assessed by the European Medicines Agency—a cross-sectional study on European Public Assessment Reports. BMC Med. **20**, 177. (10.1186/s12916-022-02377-2) 35590360 PMC9119701

[26] Naudet F, Sakarovitch C, Janiaud P, Cristea I, Fanelli D, Moher D, Ioannidis JPA. 2018 Data sharing and reanalysis of randomized controlled trials in leading biomedical journals with a full data sharing policy: Survey of studies published in the *BMJ* and *PLOS Medicine*. BMJ. **360**. (10.1136/bmj.k400) PMC5809812 29440066

[27] Dulitzki C, Crane SM, Hardwicke TE, Ioannidis JPA. 2024 Data from: Expanding the Data Ark: an attempt to make the data from highly cited social science papers publicly available. *Open Science Framework*. (https://osf.io/3kuwq/) 10.1098/rsos.240016 PMC11285638 39076822

[28] Dulitzki C, Crane SM, Hardwicke TE, Ioannidis JPA. 2024 Expanding the Data Ark: an attempt to make the data from highly cited social science papers publicly available. (10.24433/CO.5136309.v2) PMC11285638 39076822

